# Rapid and sensitive detection of Citrus Bacterial Canker by loop-mediated isothermal amplification combined with simple visual evaluation methods

**DOI:** 10.1186/1471-2180-10-176

**Published:** 2010-06-18

**Authors:** Luciano A Rigano, María R Marano, Atilio P Castagnaro, Alexandre Morais Do Amaral, Adrian A Vojnov

**Affiliations:** 1 Instituto de Ciencia y Tecnología Dr. Cesar Milstein, Fundación Pablo Cassará, Consejo Nacional de Investigaciones Científicas y Técnicas, Saladillo 2468, Ciudad de Buenos Aires, Argentina; 2 Instituto de Biología Molecular de Rosario, Departamento de Microbiología, Facultad de Ciencias, Bioquímicas y Farmacéuticas, Universidad Nacional de Rosario, Rosario, Argentina; 3 Sección de Biotecnología de la Estación Experimental Agroindustrial Obispo Colombres. UA-INSIBIO, Consejo Nacional de Investigaciones Científicas y Técnicas, Universidad Nacional de Tucumán, Las Talitas, Tucumán, Argentina; 4 Embrapa Recursos Genéticos e Biotecnologia and Centro APTA Citros Sylvio Moreira, Brasilia, AC, Cordeiropolis, Sao Paulo, Brazil

## Abstract

**Background:**

Citrus Bacterial Canker (CBC) is a major, highly contagious disease of citrus plants present in many countries in Asia, Africa and America, but not in the Mediterranean area. There are three types of Citrus Bacterial Canker, named A, B, and C that have different genotypes and posses variation in host range within citrus species. The causative agent for type A CBC is *Xanthomonas citri *subsp. *citri*, while *Xanthomonas fuscans *subsp. *aurantifolii*, strain B causes type B CBC and *Xanthomonas fuscans *subsp. *aurantifolii *strain C causes CBC type C. The early and accurate identification of those bacteria is essential for the protection of the citrus industry. Detection methods based on bacterial isolation, antibodies or polymerase chain reaction (PCR) have been developed previously; however, these approaches may be time consuming, laborious and, in the case of PCR, it requires expensive laboratory equipment. Loop-mediated isothermal amplification (LAMP), which is a novel isothermal DNA amplification technique, is sensitive, specific, fast and requires no specialized laboratory equipment.

**Results:**

A loop-mediated isothermal amplification assay for the diagnosis of Citrus Bacterial Canker (CBC-LAMP) was developed and evaluated. DNA samples were obtained from infected plants or cultured bacteria. A typical ladder-like pattern on gel electrophoresis was observed in all positive samples in contrast to the negative controls. In addition, amplification products were detected by visual inspection using SYBRGreen and using a lateral flow dipstick, eliminating the need for gel electrophoresis. The sensitivity and specificity of the assay were evaluated in different conditions and using several sample sources which included purified DNA, bacterium culture and infected plant tissue. The sensitivity of the CBC-LAMP was 10 fg of pure *Xcc *DNA, 5 CFU in culture samples and 18 CFU in samples of infected plant tissue. No cross reaction was observed with DNA of other phytopathogenic bacteria. The assay was capable of detecting CBC-causing strains from several geographical origins and pathotypes.

**Conclusions:**

The CBC-LAMP technique is a simple, fast, sensitive and specific method for the diagnosis of Citrus Bacterial Canker. This method can be useful in the phytosanitary programs of the citrus industry worldwide.

## Background

Citrus Bacterial Canker is an economic important disease in several countries, and causes great losses in fruit production and its subsidiaries [1]. There are three types of Citrus Bacterial Canker identified that have different genotypes and posses variations in host range among citrus plants. The type A CBC originating from Asia, is caused by *Xanthomonas citri *subsp. *citri*, this is the most destructive and widespread variant of the disease with a host range that includes all citrus cultivars [2]. The CBC types B and C are caused by *Xanthomonas fuscans *subsp. *aurantifolii *strains B and C, respectively. Those bacteria are limited in host range and are geographically restricted to South America. Type B CBC is present only in Argentina, Uruguay and Paraguay and is found primarily on lemon and orange [2]. Type C CBC is limited to the Sao Paulo state in Brazil and infects key or mexican lime [2]. The symptoms induced by the tree forms of canker organisms are similar and consist of cankers surrounded by chlorotic haloes and surface necrotic lesions on fruits or leaves and water-soaked lesions on leaves. Besides its leaf symptoms, this disease can cause early fruit abscission and general tree decline and the infected fruit lose market price. Moreover, quarantine restrictions are applied to prevent the spread of the pathogen to new areas, which limit drastically the trade of fresh citrus fruit with the consequent economic damage [3]. Those quarantine programs consist of rapid and reliable detection of the bacteria in all the sampled material, which include seedlings, fruits and leaves. Currently, the main procedure to detect infection is visual inspection based on disease symptoms on trees. Samples that are suspected to be positive are sent to diagnostic laboratories for further isolation on culture media. These cultures are used for reinoculation on citrus and for detection by serological methods [4]. Methodologies based on the culture of the bacterium are laborious and time consuming. In another approach, polymerase chain reaction is used for the detection of *Xcc *using different genomic portions as amplification targets [5-7]. These methods are more sensitive, specific and faster than the methods based on bacterial culture, however require modern and expensive laboratory facilities and can be difficult to perform, depending on the location. In addition, they require the use of gel electrophoresis to detect amplified products, which is long and tedious. Real-time PCR assays developed for the rapid detection of *Xcc *[4,8] have the drawback of requiring an expensive thermal cycler with a fluorescence detector.

Loop-mediated isothermal amplification (LAMP) is a recent DNA amplification technique that amplifies DNA with high specificity, efficiency and rapidity under isothermal conditions [9]. LAMP is based on the principle of autocycling strand displacement DNA synthesis performed by the *Bst *DNA polymerase, for the detection of a specific DNA sequence [9]. The technique uses four to six primers that recognize six to eight regions of the target DNA and provides very high specificity [9,10]. The technique can be carried out under isothermal conditions ranging between 60 and 65°C and produces large amounts of DNA [9]. The reaction shows high tolerance to biological contaminants [11], which can help to avoid false negative results due to the inactivation of the enzyme, a common problem in PCR. Although LAMP amplification products can also be detected by gel electrophoresis, this long procedure reduces the suitability for field applications. For this reason we used SYBRGreen I, an intercalating DNA dye, and a generic lateral flow dipstick (LFD) device to detect the positive amplification by simple visual inspection, as described previously [12-20], with potential field application. We optimized the assay for the amplification of a portion of the *pthA *gene, a well known pathogenicity determinant of CBC-causing *Xanthomonas *[21-25]. Various LAMP assays for the detection of animal and human pathogens have been developed [20,26-33], but this technique remains uncommon for bacterial plant pathogens. Here we describe a sensitive, specific, fast, and simple LAMP assay for the detection of Citrus Bacterial Canker.

## Results

**Figure 1:**
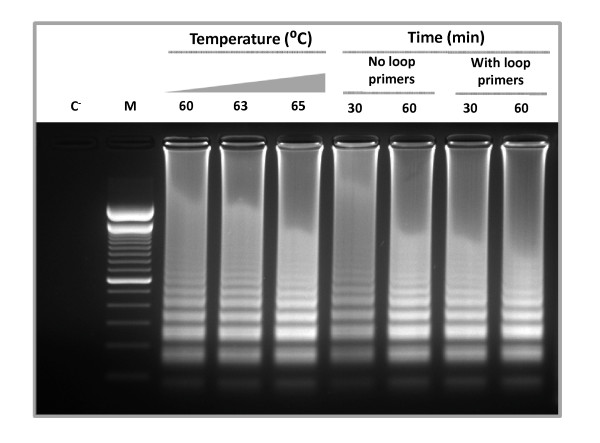
**CBC-LAMP reaction optimization**. Temperature, time and primer combinations applied to CBC-LAMP to determine the optimal reaction conditions. An aliquot of 15 μl of CBC-LAMP reaction aliquot was applied to 1.5% agarose gel electrophoresis and stained with ethidium bromide. C**^-^**: negative control without DNA. M: 100-bp DNA ladder.

**Figure 2:**
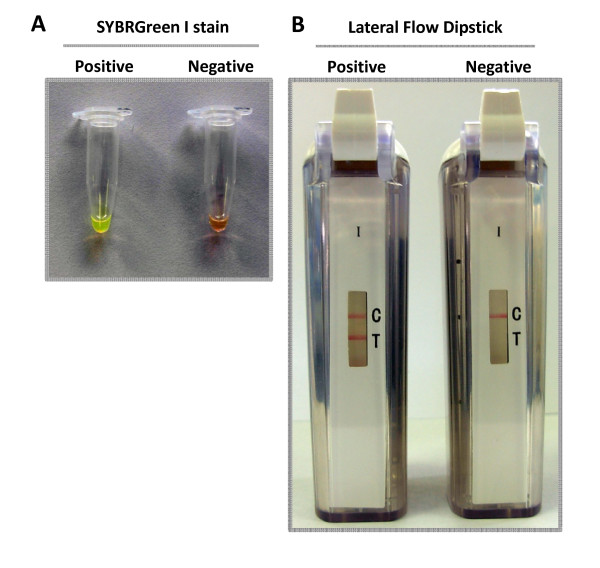
**Direct analysis of CBC-LAMP products**. Direct visual evaluation methods were used as follows. **A**-CBC-LAMP positive and negative reaction tubes were stained with SYBRGreen I and inspected under daylight. **B**-CBC-LAMP positive and negative reactions were subjected to lateral flow dipstick visual detection.

Reaction conditions were optimized to establish fast and efficient parameters for amplification. Different temperatures, times and the use of loop primers, which have the capacity to accelerate the reaction, were tested [10]. The optimal amplification of the *pthA *gene fragment was obtained at 65°C for 30 min using loop primers, as shown by agarose gel electrophoresis (Fig. 1). Amplified products exhibited a typical ladder-like pattern. No products were observed in negative control without DNA (Fig. 1). Specificity of the amplification product was confirmed by sequencing of some bands (data not shown). The samples giving positive reaction show a green color with the addition of SYBRGreen I, while the negative control remained orange (Fig. 2). The lateral flow dipstick shows two clear lines for the positive reaction (the lower line is the sample assay band and the upper one is the control line) while the negative reaction shows only the control line (Fig. 2). CBC-LAMP assays carried out using purified DNA from other common citrus and plant pathogens gave no amplification as evidenced by gel electrophoresis, SYBRGreen stain or LFD (Table 1).

**Table 1:** Specificity of CBC-LAMP assay

Species	Strain	Detection Method		
		Gel	LFD	SYBRGreen
*Xanthomonas citri *subsp. *citri *	306	+	+	+
*Xylella fastidiosa*	9a5c	-	-	-
*Candidatus *Liberibacter asiaticus	*	-	-	-
*Xanthomonas campestris *pv. *campestris *	8004	-	-	-
*Xanthomonas campestris *pv. *vesicatoria *	85-10	-	-	-
*Pseudomonas syringae*	DC3000	-	-	-
*Botrytis cinerea*	B-191	-	-	-
*Phytophthora citricola*	*	-	-	-
*Guignardia citricarpa*	*	-	-	-
*Elsinoe fawcettii*	*	-	-	-

For each dilution CBC-LAMP reaction was performed in triplicate. Gel: gel electrophoresis. LFD: lateral flow dipstick.^+^: Positive reaction.^-^: Negative reaction. * Performed with DNA from an infected plant without symptoms of CBC.

The CBC-LAMP detection limit was determined using *Xanthomonas citri *subsp. *citri *strain 306. The detection limit for *Xcc *pure DNA was 10 fg (Table 2), 5 CFU of *Xcc *cultured cells and 18 CFU from infected leave tissues according to the detection method used (Table 3). Positive amplification was obtained for every CBC-causing *Xanthomonas *strains from different regions in Argentina and around the world, including CBC types A, B and C strains. *Xanthomonas axonopodis *pv. *citrumelo*, the causative agent of Citrus Bacterial Spot, a non canker producing citrus associated bacteria, did not produced any amplification (Table 4).

**Table 2:** CBC-LAMP assay sensitivity from pure DNA

Detection method	Purified *Xanthomonas citri subsp. citri *DNA								
	**100 ng**	**10 ng**	**1 ng**	**100 pg**	**10 pg**	**1 pg**	**100 fg**	**10 fg**	**1 fg**

Gel	+	+	+	+	+	+	+	+	-
LFD	+	+	+	+	+	+	+	+	-
SYBRGreen	+	+	+	+	+	+	Nc	Nc	-

For each dilution the CBC-LAMP reaction was performed in triplicate. Gel: gel electrophoresis. LFD: lateral flow dipstick.^+^: Positive reaction.^-^: Negative reaction. Nc: The colour developed in the test tube was not clearly distinguishable between a positive or negative reaction.

**Table 3:** CBC-LAMP assay sensitivity from cultured cells and infected tissue

Strain	Specimen source	Detection method	CFU per reaction (10-fold dilutions)			
*X. citri *pv. *citri *	Pure culture		395.3	37.6	5.2	0.7
			
		Gel	+	+	+	-
		LFD	+	+	+	-
		SYBRGreen	+	+	+	-

*X. citri *pv. *citri *	Infected tissue		248.4	18.7	3.3	0.2
			
		Gel	+	+	-	-
		LFD	+	+	-	-
		SYBRGreen	+	+	-	-

For each dilution the CBC-LAMP reaction was performed in triplicate. Gel: gel electrophoresis. LFD: lateral flow dipstick. ^+^: Positive reaction.^-^: Negative reaction.

**Table 4:** Strains of Citrus pathogenic *Xanthomonas *used to evaluate the CBC-LAMP assay

Species	Strain (s)	Origin			CBC type	Detection Method		
				
		Host	Place	Country		Gel	LFD	S G
*Xanthomonas citri *subsp. *citri *	XC1CE	Tangerine	Concordia, Entre Rios	Argentina	A	+	+	+
	XC2COE	Orange	Colon, Entre Rios	Argentina	A	+	+	+
	XC3AM-1, XC3AM-2	Lemon	Apostoles, Misiones	Argentina	A	+	+	+
	XC4PM	Grapefruit	Posadas, Misiones	Argentina	A	+	+	+
	XC5LF-1, XC5LF-2	Grapefruit	Las Lomitas	Argentina	A	+	+	+
	XC7ETS-1, XC7ETS-2	Orange	El Tabacal, Salta	Argentina	A	+	+	+
	XC8SPB-1, XC8SPB-2	Orange	San Pedro, Buenos Aires	Argentina	A	+	+	+
	XC9CAT -1, XC9CAT-2	Orange	Catamarca	Argentina	A	+	+	+
	XC10BVC -1, XC10BVC -2	Lemon	Bella Vista, Corrientes	Argentina	A	+	+	+
	XC10BVC -3, XC10BVC -4, XC10BVC -5	Orange	Bella Vista, Corrientes	Argentina	A	+	+	+
	XC10BVC -6, XC10BVC -7	Grapefruit	Bella Vista, Corrientes	Argentina	A	+	+	+
	XC10BVC-8	Tangerine	Bella Vista, Corrientes	Argentina	A	+	+	+
	XC6FT-1, XC6FT-2, XCT2, XCT3, XCT7, XCT9, XCT18, XCT22, XCT31, XCT33, XCT42	Lemon Leave	Tucumán	Argentina	A	+	+	+
	XCT1, XCT17, XCT19, XCT21, XCT28, XCT29,	Lemon Fruit	Tucumán	Argentina	A	+	+	+
	XCT44	Tangerine Leave	Tucumán	Argentina	A	+	+	+
	306 (sequenced strain)	--	--	Brazil	A	+	+	+
	625	--	Aratiba, Sao Paulo	Brazil	A	+	+	+
	1637	--	Embaúba, Sao Paulo	Brazil	A	+	+	+
	1740	--	--	China	A	+	+	+
	1801	--	--	Oman	A*	+	+	+
*Xanthomonas fuscans *subsp. *Aurantifolii*	B832	--	--	Argentina	B	+	+	+
	382,1473	--	--	Brazil	C	+	+	+
*Xanthomonas axonopodis *pv. *Citrumelo*	1925	--	--	USA	--	-	-	-

For each isolate CBC-LAMP reaction was performed in triplicate. When available, detailed data about the place of origin and type of sample is included. Gel: gel electrophoresis. LFD: lateral flow dipstick. SG: SYBRGreen.^+^: Positive reaction.^-^: Negative reaction.

The potential use of this technique in location was evaluated. Infected lemon and orange fruits and leaves were collected in field. All the field samples with canker symptoms gave positive reaction using all amplicon detection methods presented in this work (Additional file 1 fig. S1).

## Discussion

Citrus Bacterial Canker is a serious, aggressive disease that attacks most species of citrus worldwide. Rapid and correct diagnosis of the pathogens is crucial to minimize and control damage to the citrus industry. During the last decade several nucleic acid amplification-based methods have been developed for the detection of CBC causing-*Xanthomonas *[4-8]. These methods are fast, specific and sensitive, but are not applicable for field trials, since they can require equipment and facilities that are not easily portable. The methodology presented in this work demonstrated that CBC can be detected quickly, sensitively and specifically by using LAMP technology in an equipment-free procedure. The use of gene *pthA *was proven to be very selective and efficient for the diagnosis of CBC as it has been described previously [4,6,8]. Our studies suggest that the sensitivity could be greater than in those achieved previously by conventional PCR [5,7] and comparable to those reported by using real-time PCR [4], although a comparative study must be performed to confirm it. On the other hand, the high sensitivity observed in this assay could require special attention in order to avoid final product contamination, a common setback in DNA-based diagnostics.

Specificity studies found no cross reaction with citrus and other plant pathogens, due to the fact that LAMP recognizes several sites in the template, improving specificity over conventional methods such as PCR [9]. Furthermore, a negative result was obtained with *Xanthomonas citri *pv. *citrumelo*, a closely related, non canker inducing *Xanthomonas*, ethiological agent of Citrus Bacterial Spot. This is concurrent with the fact that no *pthA *homolog has been found in this bacterium [6].

A BLAST search using as a query the target sequence of CBC-LAMP shows high identity with different CBC-causing *Xanthomonas *strains. Because *pthA *belongs to a family of *Xanthomonas *avirulence-pathogenicity genes, some grade of identity is found with other *Xanthomonas *spp., however as this xanthomonads do not attack citrus, this should not be a problem in diagnosing and identifying CBC, as discussed by Cubero and Graham in a previous study [6]. Positive reaction was obtained in all *Xanthomonas citri *subsp*, citri *type A strains tested, comprising reference strains and field isolates from Argentina and other countries. Interestingly the strain A*, a variant of the A strains from southwest Asia [4] is also recognized by the assay. Furthermore, CBC-LAMP was effective in the detection of type B and C strains; these results and the positive results obtained with field samples from lemon and orange confirm the robustness of the method here described for diagnosis of Citrus Bacterial Canker whatever the infecting variant is.

The DNA extraction method using Chelex allowed a fast and efficient DNA extraction from citrus plants infected with *Xcc *as described previously [4]. This method of sample preparation can be useful to shorten the time required in sample processing and in reducing the need for equipment. Amplicon detection by visual methods proved to be as sensitive as the gel in the case of lateral flow dipstick, but much faster and convenient. In the case of detection by adding SYBRGreen, when working with low concentrations of DNA the difference between positive and negative samples were not clear, this evidence a loss of sensitivity using the SYBRGreen detection method. Indeed we found the detection of the amplicon more robust using the lateral flow dipstick methodology as compared to the use of SYBRGreen.

The selected optimal conditions for amplification according to the experiments were 65°C for 30 minutes and with the addition of loop primers, which accelerated the reaction by hybridization to the stem loops as described previously [10]. The combination of an isothermal amplification reaction followed by a visual detection method allows the detection of this pathogen with a speed not reported so far. The time it takes to perform the test using the lateral flow dipstick is approximately 45 min including the detection of the amplification product, without DNA preparation. This speed of detection coupled with the ability to be conducted in the field can be very important in plant protection programs for citrus producers and importer countries.

## Conclusions

Considering the data from the loop-mediated isothermal amplification assay combined with the lateral flow dipstick device, we conclude that the technique is specific, reliable, sensitive, fast and represents a powerful diagnostic tool for CBC. The CBC-LAMP assay requires only a simple water bath, which makes this technique suitable as a field diagnosis tool in locations where more complex laboratory equipment is not available.

## Methods

### Bacterial strains

*Xanthomonas citri *subsp. *citri *strain 306 [34] was the reference strain used in this study; in addition, field isolates of *Xcc *from several geographical origins and different pathotypes were tested. The strains used in this work belong to the strain collection of the Dr. Canteros' laboratory at Instituto Nacional de Tecnología Agropecuaria (INTA), Bella Vista, Corrientes, Argentina. All the strains were propagated on their specific medium at 28°C.

### Infected Plant Tissue

For sensitivity tests, we used *C. limón *cv. Eureka leaves artificially inoculated with *Xcc *strain 306 as described previously [35]. Lemon and orange field samples were collected from citrus orchards in Tucumán province in Argentina from plants positives for CBC.

### DNA extraction

For sensitivity with pure DNA and specificity assays, DNA was extracted using the Wizard^® ^Genomic DNA purification Kit, Promega, Madison, WI, USA, according the manufacturer instructions. DNA obtained from cultured bacteria and infected tissue were purified using Chelex^® ^100 resin, Biorad, Hercules, CA, USA, as described previously [4].

### LAMP reaction

**Figure 3:**
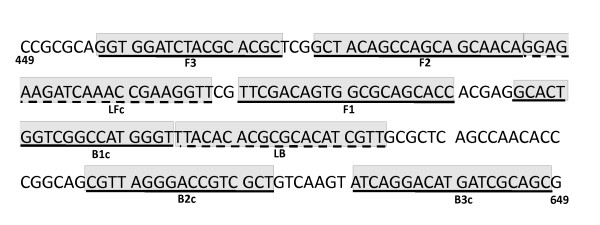
**Localization of target sequences used for primer construction**. Target sequences used for LAMP primer design are underlined and shadowed. The figure shows a portion of *pthA *gene from *Xcc *449 nucleotides downstream from the start codon.

Oligonucleotide LAMP primers were designed according to the published sequence of *PthA4 *gene from *Xcc *[GenBank: XACb0065] using the program Primer Explorer version 4 (Net Laboratory, Tokyo, Japan) targeting the 5'-end region of the gene (Fig. 3) which generated the primers XCC-F3, XCC-B3, XCC-FIP and XCC-BIP (Table 5). In addition a set of two Loop primers, XCC-LF and XCC-LB was generated for reaction acceleration (Table 5). LAMP assay was performed using a thermal dry block with a 0.5-mL PCR tube holder. Several reaction conditions were assayed, including different temperature, time (Fig. 1), and primer concentrations (data not shown). The final LAMP conditions comprised 40 pmol each of primers XCC-FIP and XCC-BIP, 5 pmol each of outer primers XCC-F3 and XCC-B3, 20 pmol each of loop primers XCC-LF and XCC-LB, 8 U of *Bst *DNA polymerase, 4.5 mM MgSO_4_, 1.4 mM of dNTP mix, 20 mM Tris-HCl (pH 8.8), 10 mM KCl, 10 mM (NH_4_)_2_SO_4_, 0.1% Triton X-100 and 1.6 M betaine, in a final volume of 25 μL containing the template. This mixture was incubated at 65°C for 30 minutes.

**Table 5:** Sequences of primers used for CBC-LAMP assay

Primer Name	Type	Sequence (5'-3')	Length
XCC-F3	F3	GGTGGATCTACGCACGC	17 mer
XCC-B3	B3	GCTGCGATCATGTCCTGAT	19 mer
XCC-FIP	FIP (F1c+F2)	GGTGCTGCGCCACTGTCGAA - GCTACAGCCAGCAGCAACA	39 mer
XCC-BIP	BIP (B1c+B2)	GCACTGGTCGGCCATGGGTA - GCGACGGTCCCTAACG	36 mer
XCC-LF	LF	AACCTTCGGTTTGATCTTCTCC	22 mer
XCC-LB	LB	TTACACACGCGCACATCGT	19 mer

### Analysis of LAMP products

The amplified products were subjected to electrophoresis at 100V for 50 minutes on a 1.5% agarose gel, followed by ethidium bromide staining. To confirm the specificity of the product some bands were cut and sequenced (data not shown). The sequences obtained were used as queries to perform BLAST searches [36] in order to confirm identity.

### Direct analysis of LAMP products

For direct analysis of LAMP products, generic LFD strips (Type I BEST™ cassette, Biohelix Co, Beverly, MA, USA) were used. These strips are capable to detect an amplicon that is dual labelled with biotin and fluorescein. For this purpose we used 5'-biotin-labelled XCC-FIP primers and 5'-fluorescein-labelled XCC-BIP primers in the amplification reactions. The labelled oligonucleotides were purchased from Integrated DNA Technologies™ requesting HPLC purification. After amplification reaction, the reaction tube is inserted in the detection chamber; the dual tagged amplicon is automatically mixed with the detection buffer, and directed by capillary flow to the strip. The amplicon is detected in the test zone (T) of the cassette whereas the control zone (C) serves as a control for the flow function. The complete detection process takes about 10 minutes. More information is available in the manufacturer web site http://www.biohelix.com/products/Type_I_&_Type_II_Cassettes.asp.

The inspection for amplification was also performed through observation of colour change after addition of 1 μL of a 1:1000 dilution of SYBRGreen dye to the reaction tube. In the case of positive amplification the original orange color of the dye turns to green which can be examined in daylight.

### Sensitivity of LAMP

In the sensitivity assay from pure DNA, 100 ng of *Xcc *DNA was 10-fold diluted and used as template for LAMP amplifications. For sensitivity assay from cultured cells, 10-fold dilutions of *Xcc *liquid culture were prepared, then a 4- μL aliquot from the dilutions were used for plating and colonies enumeration and, after DNA purification processing, 4-μL aliquots of dilutions were used as DNA template. In the assay for sensitivity from infected tissue, artificially-infected citrus leaves were used as starting material for the same procedure mentioned above.

## Authors' contributions

LAR designed the experiments, performed the experimental work and drafted the manuscript; MRM and APC contributed to coordinate the study and to draft the manuscript; AMDA isolated the DNA sample from *Candidatus *Liberibacter asiaticus used for specificity tests and critically revised the manuscript; AAV participated in the analysis and interpretation of the data and prepared the final version of the manuscript. All authors read and approved the final version of the manuscript.

## Supplementary Material

Additional file 1 **Fig. S1 CBC-LAMP performance with field samples**. Field samples of Lemon and Orange was collected and analyzed by CBC-LAMP. LFD: lateral flow dipstick. SG: SYBRGreen. GEL: gel electrophoresis. Click here for file
