# Supplementary material for: Rapid and sensitive detection of Citrus Bacterial Canker by loop-mediated isothermal amplification combined with simple visual evaluation methods
Source: BMC Microbiol. 2010 Jun 18;10:176. doi: 10.1186/1471-2180-10-176 (PMC2895605; doi:10.1186/1471-2180-10-176)
Supplement: Additional file 1 — Fig. S1 CBC-LAMP performance with field samples. Field samples of Lemon and Orange was collected and analyzed by CBC-LAMP. LFD: lateral flow dipstick. SG: SYBRGreen. GEL: gel electrophoresis. [file 1471-2180-10-176-S1.PPT]

## Slide 1
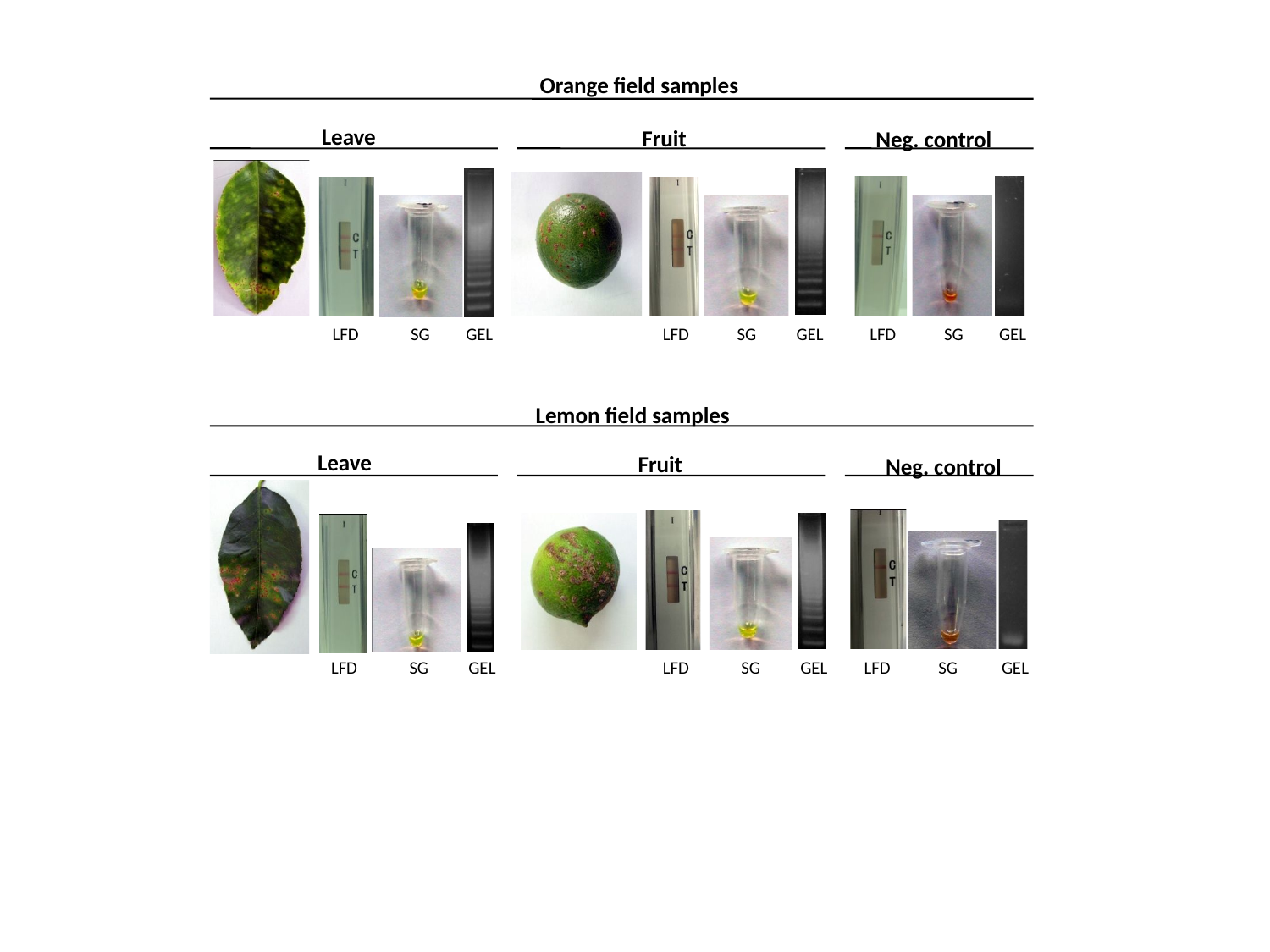

Orange field samples
Leave
Fruit
Neg. control
 LFD SG GEL
 LFD SG GEL
 LFD SG GEL
Lemon field samples
Leave
Fruit
Neg. control
 LFD SG GEL
 LFD SG GEL
 LFD SG GEL
